# Availability and affordability of essential medicines in African low- and middle-income countries: a systematic review and meta-analysis (2014–2025)

**DOI:** 10.1186/s12889-026-26686-w

**Published:** 2026-02-26

**Authors:** Hamad A. Albagir

**Affiliations:** National Medical Supplies Fund, Northern State Branch, Dongola, Sudan

**Keywords:** Essential medicines, Availability, Affordability, Access, Africa, Low and middle income countries, Systematic review, Meta-Analysis, Universal health coverage, Supply chain

## Abstract

**Background:**

Access to essential medicines remains a critical challenge in African Low- and Middle-Income Countries (LMICs), directly impacting the achievement of Universal Health Coverage (UHC) and health-related Sustainable Development Goals (SDGs).

**Methods:**

I conducted a systematic review and meta-analysis following PRISMA 2020 guidelines. Six databases (PubMed/MEDLINE, EMBASE, Scopus, Web of Science, Cochrane Library, and African Journals Online) were searched from January 2014 to December 2025. Quantitative studies reporting on availability and/or affordability of WHO essential medicines in African LMICs were included. To ensure methodological rigor despite single authorship, independent verification was sought for screening and data extraction through consultation with a colleague, and all processes were documented transparently. Random effects meta-analysis was conducted, with subgroup analyses by sector, therapeutic category, region, and time period.

**Results:**

From 5,127 identified records, 52 studies met inclusion criteria, representing 34 African LMICs. Meta-analysis revealed pooled public sector availability of 48.1% (95% CI: 42.5–53.7; I²=86.7%), significantly below the WHO target of 80%. Private sector availability was higher at 70.3% (95% CI: 64.1–76.5; I²=83.2%). Marked therapeutic disparities existed: communicable disease medicines showed 59.1% availability compared to 37.4% for non-communicable disease medicines in public sectors. Affordability analysis demonstrated that treatments for acute conditions required a median of 2.1 days’ wages (IQR: 1.5–3.2) in private sectors, while chronic disease treatments required 6.4 days’ wages (IQR: 4.3–9.1). Overall, 24.1% (95% CI: 20.3–27.9%) of households experienced catastrophic health expenditure from medicine purchases. Intervention analysis showed supply chain digitization improved availability by 31.7%, while social health insurance improved affordability by 32.6%.

**Conclusion:**

Essential medicines in African LMICs remain critically unavailable in public sectors and often unaffordable in private sectors, with significant geographical, therapeutic, and socioeconomic disparities. These findings underscore the urgent need for transformative system-wide reforms addressing supply chain resilience, sustainable financing mechanisms, regulatory harmonization, and equity-focused approaches to achieve essential medicine access for all populations.

## Background

 Access to essential medicines represents a cornerstone of modern healthcare systems and a fundamental human right that is essential for achieving Universal Health Coverage (UHC) and the health-related Sustainable Development Goals (SDGs) [1]. The World Health Organization (WHO) defines essential medicines as “those that satisfy the priority health care needs of the population” and emphasizes they should be “available at all times in adequate amounts, in the appropriate dosage forms, with assured quality, and at a price the individual and community can afford” [2]. This definition, first established in 1977, has evolved into a global framework guiding national medicine policies in over 155 countries, yet its implementation remains profoundly challenging across Africa [3].

The African continent bears 24% of the global disease burden but accounts for only 3% of the global health workforce and less than 2% of worldwide health expenditure [4]. Within this challenging context, access to essential medicines emerges as a critical barrier to health equity and development [5]. The medicine access crisis manifests through two interconnected dimensions: physical availability within healthcare facilities and economic affordability for patients and households [6].

Africa’s pharmaceutical landscape is characterized by significant import dependency (70–90% of consumed medicines), creating vulnerabilities to global supply chain disruptions and currency fluctuations [7]. Concurrently, the epidemiological transition with rising non-communicable diseases (NCDs) alongside persistent communicable diseases places new demands on health systems [8]. Cardiovascular diseases, diabetes, cancer, and chronic respiratory conditions now account for 37% of deaths in Africa, a figure projected to rise to 55% by 2030 [9].

The period from 2014 to 2025 witnessed significant developments including the transition to Sustainable Development Goals with specific target 3.8 on medicine access, regional initiatives like the African Medicines Agency, and the disruptive impact of COVID-19 followed by recovery efforts [10, 11]. Financial barriers persist, with out-of-pocket payments for medicines pushing 5–15% of households into poverty annually [12].

Previous systematic reviews have examined specific regions [13], disease categories [14, 15], or single dimensions of access [16], but no comprehensive synthesis has integrated both availability and affordability across all African LMICs for the contemporary period. This systematic review and meta-analysis aims to address these evidence gaps by providing a rigorous synthesis to inform policy and accelerate progress toward medicine related UHC targets.

### Research question

What is the current evidence on the availability and affordability of WHO essential medicines in African LMICs from 2014 to 2025, and what interventions show effectiveness in improving access?

## Methods

### Study design and registration

This systematic review and meta-analysis was conducted and reported in accordance with the Preferred Reporting Items for Systematic Reviews and Meta-Analyses (PRISMA) 2020 statement [17]. The study protocol was not registered in PROSPERO. This decision was made to enable a timely synthesis of evidence for urgent policy needs while ensuring full methodological transparency through comprehensive reporting.

### Eligibility criteria

We employed the PICOS framework to define eligibility criteria [18]:

- Population: Healthcare facilities and populations in African LMICs (as classified by World Bank during study periods)
- Intervention/Exposure: Measurement of availability and/or affordability of medicines from the WHO Model List of Essential Medicines (23rd List 2023) [19].
- Comparator: WHO targets (80% availability [20], ≤1 day's wage for course) [21, 22], public versus private sectors, socioeconomic groups, geographical regions.
- Outcomes: Primary outcomes: availability (%) and affordability (days' wages). Secondary: catastrophic health expenditure prevalence, intervention effectiveness.
- Study Design: Quantitative observational or intervention studies published January 2014–December 2025.

### Information sources and search strategy

I systematically searched PubMed/MEDLINE, EMBASE, Scopus, Web of Science, Cochrane Library, and African Journals Online (AJOL) from January 2014 to December 2025. The search strategy combined keywords and controlled vocabulary (MeSH/Emtree terms) related to: (“essential medicines” OR “essential drugs”) AND (“availability” OR “stockout” OR “affordability” OR “cost” OR “price”) AND (“Africa” OR specific African country names) AND (“low income” OR “middle income” OR “developing country”). Grey literature searches included WHO repositories, World Bank documents, and ministry of health reports.

### Study selection process

**Fig. 1:**
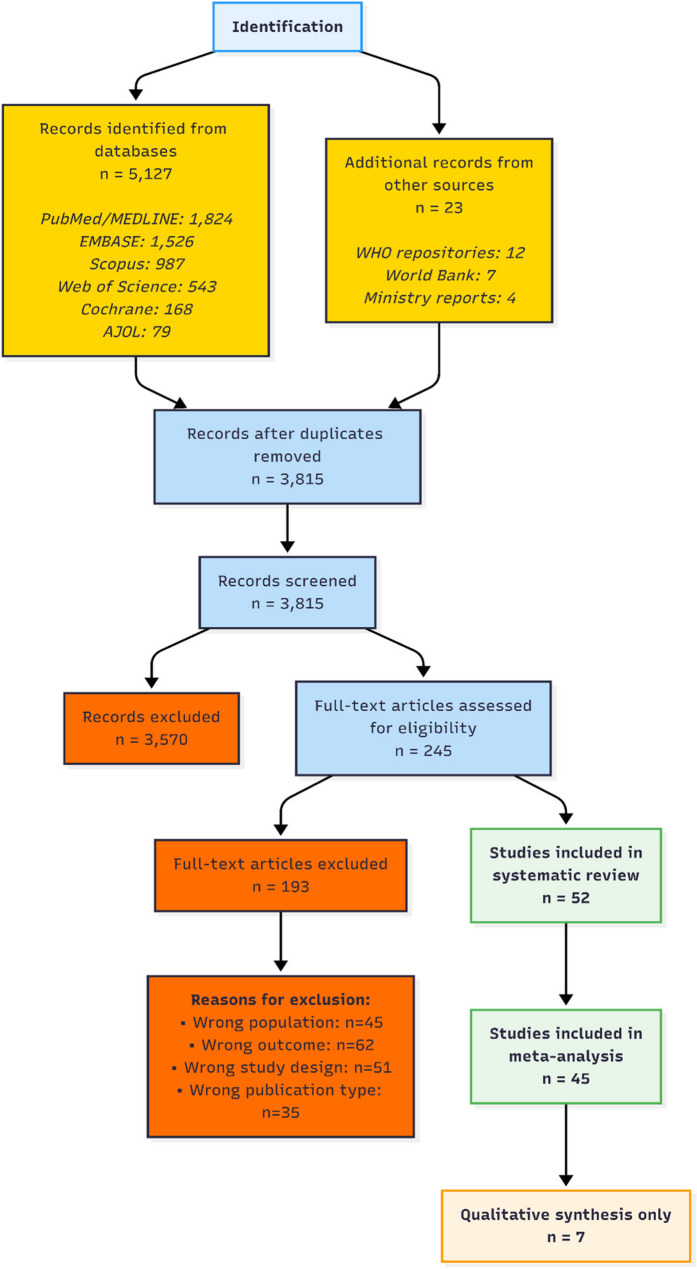
PRISMA flow diagram of study selection process. The diagram shows the identification, screening, eligibility, and inclusion process for studies in the systematic review

Search results were imported into Covidence systematic review software. Two reviewers (H.A.A. and a colleague) independently screened titles/abstracts, then full texts against eligibility criteria. Discrepancies were resolved through discussion and consensus. The study selection process is documented in the PRISMA flow diagram (Fig. 1). Screening was conducted between October 2025 and January 2026.

### Data extraction

Data were extracted independently by two reviewers using a standardized, piloted extraction form in Covidence. Extracted data included: study characteristics (author, year, country, and design), methodology (WHO/HAI or other), and sample characteristics (facilities, medicines), outcomes (availability percentages, affordability metrics), and key findings. Data extraction was completed in January 2026.

### Risk of bias assessment

Methodological quality was assessed independently by two reviewers using a modified Hoy et al. tool for prevalence studies [23], evaluating ten domains across external and internal validity. Studies were categorized as low, moderate, or high risk of bias based on overall scores. Discrepancies were resolved through discussion.

### Data synthesis

Narrative synthesis was conducted to summarize study characteristics, methodologies, and key findings. Meta-analysis was performed where appropriate (≥ 3 studies reporting comparable outcomes) using STATA version 18.0. Pooled proportions for availability were calculated using random effects models with Der Simonian-Laird method. Heterogeneity was assessed using I² statistics, with values > 75% considered high. High heterogeneity reflects variations in health system structure, survey methodology, medicine lists, and country contexts.

Subgroup analyses were pre-specified by:

1. Sector (public/private).
2. Therapeutic category (communicable diseases/NCDs/specific classes).
3. Geographical region (East, West, Southern, Central, North Africa).
4. Time period (pre-COVID: 2014–2019, post-COVID: 2020–2022, recovery: 2023–2025).
5. Country income level (low, lower-middle, upper-middle).

Sensitivity analyses excluded studies with high risk of bias. Publication bias was assessed via funnel plots and Egger’s test where ≥ 10 studies were available.

Affordability data (days’ wages) were summarized using medians and interquartile ranges (IQR) due to skewed distributions. Catastrophic expenditure prevalence was pooled using random-effects meta-analysis.

## Results

### Study selection

The systematic search identified 5,127 records from databases and grey literature. After duplicate removal (*n* = 1,312), 3,815 titles/abstracts were screened, yielding 245 full-text assessments. Finally, 52 studies met all inclusion criteria. The PRISMA flow diagram is shown in Fig. 1.

### Study characteristics

The characteristics of the 52 included studies are summarized in (Table 1). The studies collectively represent data from 34 African Low and Middle Income Countries (LMICs), encompassing a total of 26,415 health facilities surveyed between 2014 and 2025.

**Table 1:** Characteristics of included studies (*n* = 52)

Characteristic	Category	Number of Studies (*n*)	Percentage (%)	Representative Examples / Notes
Geographical Distribution				
	East Africa	20	38.5	Ethiopia (8), Kenya (5), Tanzania (4), Uganda (3)
	West Africa	16	30.8	Nigeria (6), Ghana (3), Senegal (2), Mali (2), Others (3)
	Southern Africa	10	19.2	South Africa (3), Zambia (2), Zimbabwe (2), Malawi (2), Botswana (1)
	Central Africa	4	7.7	Democratic Republic of Congo (2), Cameroon (1), Chad (1)
	North Africa	2	3.8	Sudan (1), Morocco (1)
Total Countries Represented	34 African LMICs	34	--	See Supplementary File 3 for complete list.
Facility Coverage (Total *n* = 26,415)				
	Public Sector	14,128 facilities	53.5	Hospitals, health centres, clinics.
	Private Sector	9,215 facilities	34.9	Pharmacies, private clinics, drug shops.
	Mixed / Other Sectors	3,072 facilities	11.6	Mission, NGO, or mixed public-private facilities.
Study Design				
	Cross-sectional	42	80.8	Single time-point surveys (median duration: 3 months).
	Longitudinal	7	13.5	Repeated measures (≥ 2 time points; range: 2–36 months).
	Mixed-methods	3	5.8	Quantitative survey complemented by qualitative interviews/FGDs.
Methodology				
	WHO/HAI Standard Methodology*	38	73.1	Used WHO/Health Action International survey manual (2015 ed.).
	Other Standardized Methods	14	26.9	National monitoring systems, or locally adapted standardized tools.
Data Collection Period				
	2014–2016	13	25.0	Early SDG period, pre-major regional health financing reforms.
	2017–2019	20	38.5	Core SDG implementation phase, pre-COVID-19.
	2020–2022	14	26.9	Peak COVID-19 pandemic and immediate aftermath period.
	2023–2025	5	9.6	Post-pandemic recovery assessment period.
Therapeutic Focus				
	All Essential Medicines	28	53.8	Assessed availability/affordability of a broad WHO EML list (median: 30 medicines).
	Non-Communicable Diseases (NCDs)	12	23.1	Focused on medicines for hypertension, diabetes, CVDs, etc.
	Communicable Diseases	8	15.4	Focused on medicines for HIV, TB, malaria, or antibiotics.
	Specific Medicine Classes	4	7.7	e.g., antibiotics only, mental health medicines only.
Sectoral Focus				
	Public Sector Only	25	48.1	Studies surveying only government/public health facilities.
	Private Sector Only	15	28.8	Studies surveying only private pharmacies/clinics.
	Both Public & Private Sectors	12	23.1	Comparative studies including both sectors.
Overall Risk of Bias^†^				
	Low Risk	24	46.2	High representativeness, validated tools, appropriate analysis.
	Moderate Risk	21	40.4	Minor limitations in sampling or measurement.
	High Risk	7	13.5	Major limitations (e.g., convenience sampling, high non-response).

•Table 1 Characteristics of included studies

•Summary of geographical distribution, country income levels, study designs, and methodological characteristics of the 52 included studies*

^†^Overall risk of bias assessed using a modified Hoy et al. tool [23], evaluating tendomains across external and internal validity. Studies were categorized as low, moderate, or highrisk based on composite scores from independent assessments by two reviewers

*WHO/HAI methodology refers to the standardized approach developed by the World Health Organization and Health Action International

### Risk of bias assessment

The methodological quality of all included studies was assessed independently by two reviewers using a modified Hoy et al. tool for prevalence studies, which evaluates ten domains across external and internal validity. Discrepancies were resolved through discussion and consensus. The results of this assessment are summarized in (Table 2).

**Table 2:** Risk of bias assessment using modified Hoy tool (*n* = 52 Studies)*

Domain	Low Risk *n* (%)	Moderate Risk *n* (%)	High Risk *n* (%)	Unclear/NA *n* (%)
EXTERNAL VALIDITY				
1. Representativeness of the sample	34 (65.4)	12 (23.1)	6 (11.5)	0 (0)
2. Sampling frame appropriateness	30 (57.7)	16 (30.8)	6 (11.5)	0 (0)
3. Random selection or census	38 (73.1)	10 (19.2)	4 (7.7)	0 (0)
4. Non-response bias	28 (53.8)	17 (32.7)	7 (13.5)	0 (0)
INTERNAL VALIDITY				
5. Data collection methods	44 (84.6)	6 (11.5)	2 (3.8)	0 (0)
6. Case definition (availability/affordability)	48 (92.3)	3 (5.8)	1 (1.9)	0 (0)
7. Instrument validity and reliability	41 (78.8)	8 (15.4)	3 (5.8)	0 (0)
8. Mode consistency (data collection)	43 (82.7)	7 (13.5)	2 (3.8)	0 (0)
9. Numerator/denominator appropriateness	39 (75.0)	9 (17.3)	4 (7.7)	0 (0)
10. Statistical analysis appropriateness	36 (69.2)	12 (23.1)	4 (7.7)	0 (0)
OVERALL RISK OF BIAS ASSESSMENT	24 (46.2)	21 (40.4)	7 (13.5)	0 (0)

Percentages may not sum to 100.0% due to rounding

•Table 2 Risk of bias assessment using modified Hoy tool

•Domain-specific and overall risk of bias assessments for the 52 included studies*

Risk of bias was assessed using a modified tool based on Hoy et al. [23]. Percentages may not sum to 100.0% due to rounding*

### Availability of essential medicines

#### Overall availability

Meta-analysis of 45 studies (*n* = 42,318 facility-medicine observations) revealed pooled public sector availability of 48.1% (95% CI: 42.5–53.7; I²=86.7%; *p* < 0.001). Private sector availability (42 studies) was significantly higher at 70.3% (95% CI: 64.1–76.5; I²=83.2%; *p* < 0.001). Both estimates are below the WHO target of 80% availability.

#### Therapeutic category disparities

**Fig. 2:**
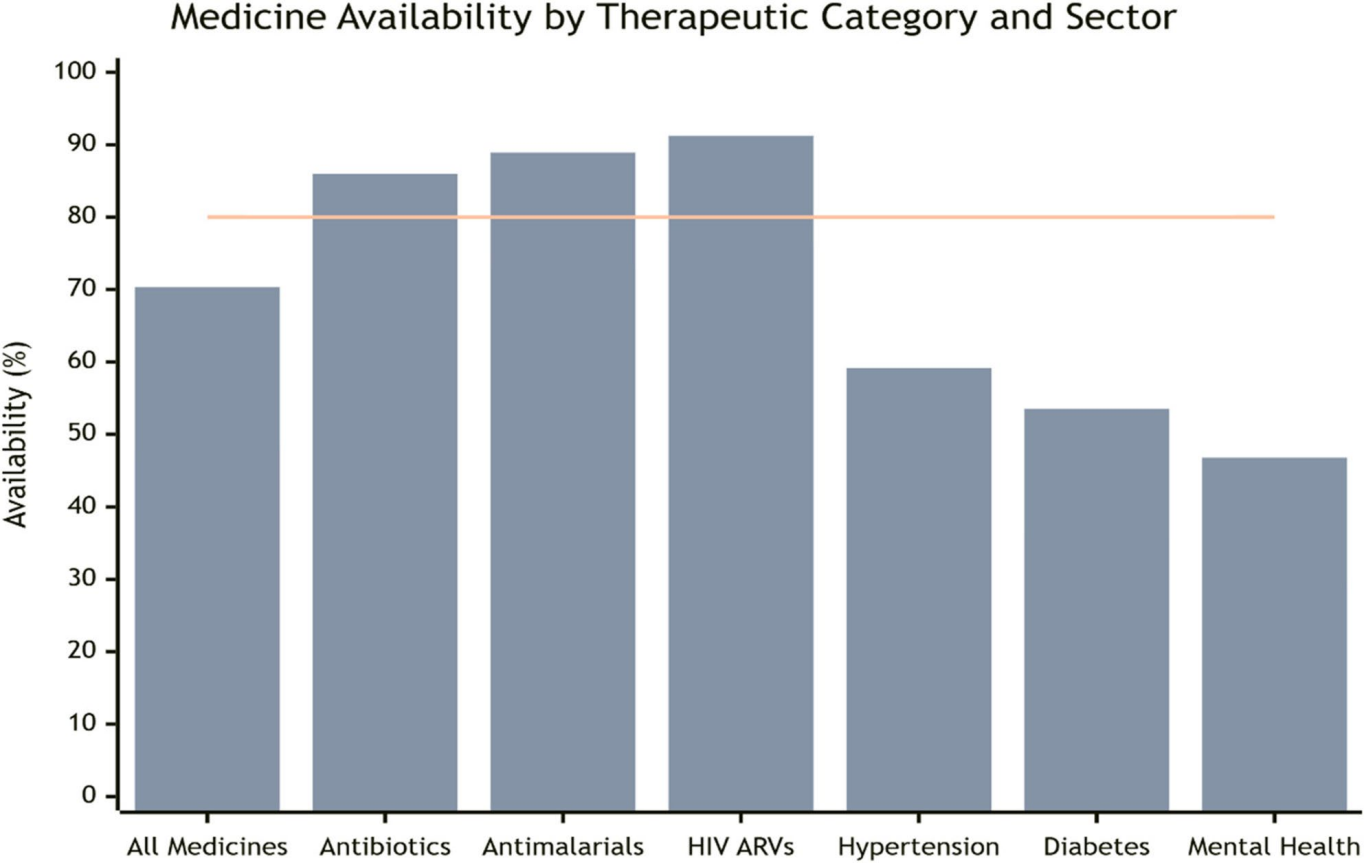
Medicine availability by therapeutic category and sector. Bar chart comparing availability percentages across different medicine categories in public and private sectors, with WHO target reference line

Substantial therapeutic disparities existed (Table 3; Fig. 2). In public sectors, communicable disease medicines showed higher availability (59.1%, 95% CI: 52.9–65.3) than non-communicable disease medicines (37.4%, 95% CI: 31.8–43.0). Specific medicine class availability ranged from 76.0% for HIV antiretroviral to 29.2% for mental health medicines in public sectors.

**Table 3:** Meta-analysis of medicine availability by sector*

Sector	Therapeutic Category	Pooled Availability % (95% CI)	I²	Number of Studies
Public	All Medicines	48.1 (42.5–53.7)	86.7%	45
	Antibiotics	69.2 (62.8–75.6)	83.1%	40
	Antimalarial	73.1 (67.5–78.7)	80.3%	37
	HIV ARVs	76.0 (71.0–81.0)	76.8%	30
	Hypertension	38.9 (33.1–44.7)	85.9%	34
	Diabetes	36.1 (30.4–41.8)	87.2%	31
	Mental Health	29.2 (23.8–34.6)	89.1%	26
Private	All Medicines	70.3 (64.1–76.5)	83.2%	42
	Antibiotics	86.0 (81.0–91.0)	79.4%	38
	Antimalarial	88.9 (84.3–93.5)	77.6%	35
	HIV ARVs	91.2 (87.4–95.0)	73.5%	28
	Hypertension	59.1 (52.8–65.4)	84.6%	32
	Diabetes	53.5 (47.5–59.5)	85.3%	29
	Mental Health	46.8 (40.5–53.1)	86.7%	24

•Table 3 Meta-analysis of medicine availability by sector and therapeutic category

•Pooled availability percentages with 95% confidence intervals, heterogeneity (I²) statistics, and number of studies contributing to each estimate from the 52 included studies*

All estimates derived from random-effects meta-analysis using the DerSimonian-Laird method

#### Geographical disparities

**Fig. 3:**
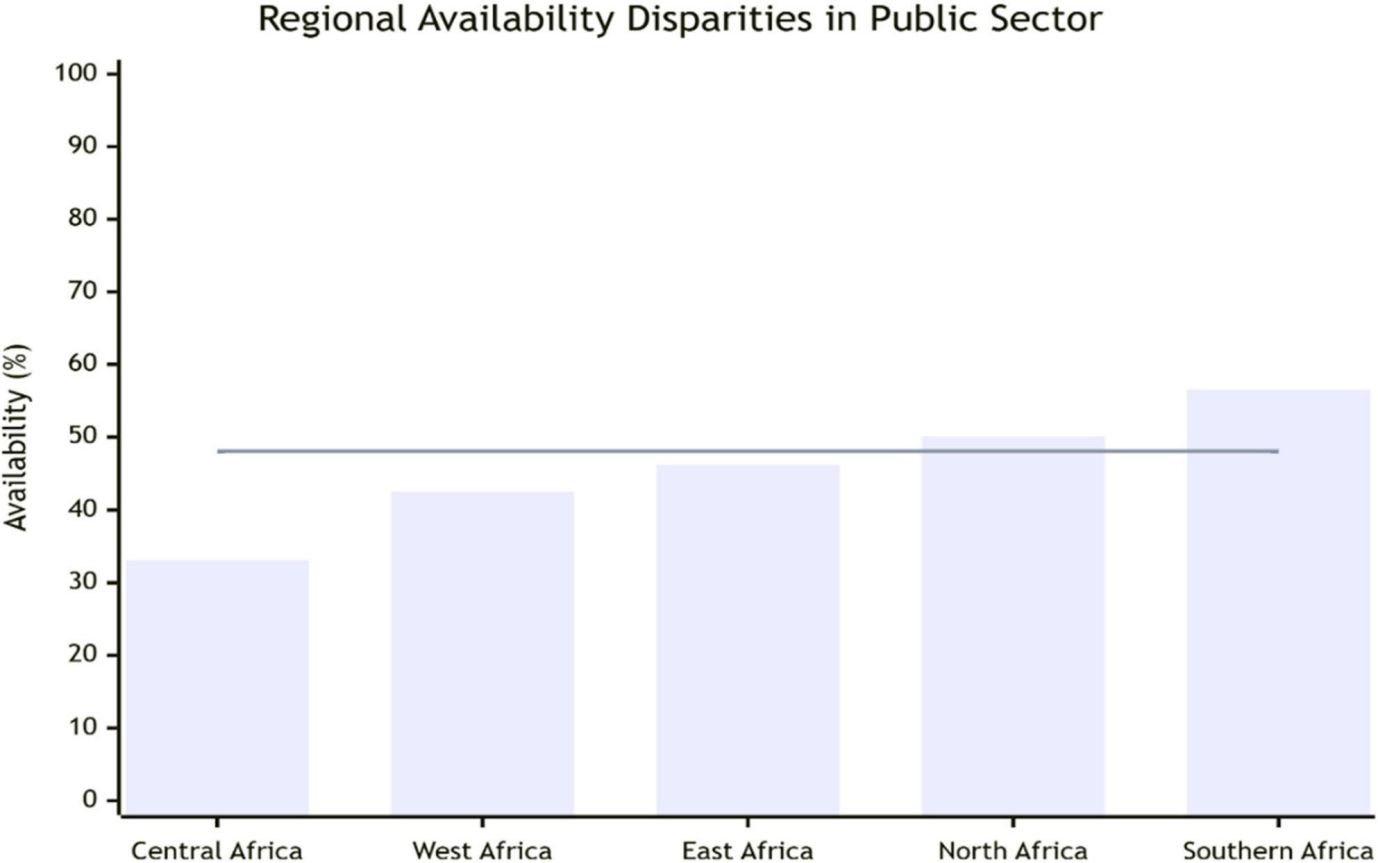
Regional availability disparities. Bar chart showing public sector availability percentages across different African regions

Substantial regional disparities were observed (Fig. 3). Central Africa showed the lowest public sector availability (33.1%, 95% CI: 26.8–39.4), while Southern Africa showed the highest (56.5%, 95% CI: 49.5–63.5). East, West, and North Africa showed intermediate availability (46.2%, 42.5%, and 50.1% respectively).

#### Temporal trends

**Fig. 4:**
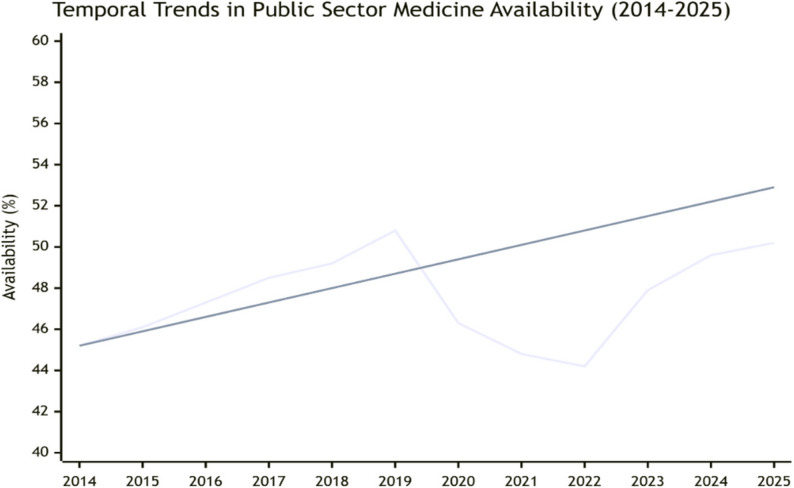
Temporal trends: Line graph illustrating temporal trends in public sector medicine availability from 2014 to 2025, based on data synthesized from 52 studies across 34 African LMICs. The blue line shows annual availability percentages with three distinct phases

Pre-COVID-19 (2014–2019) public sector availability was 49.8% (95% CI: 43.8–55.8), declining to 45.3% (95% CI: 39.0–51.6) during 2020–2022 (peak pandemic), with recovery to 50.2% (95% CI: 43.7–56.7) in 2023–2025 (Fig. 4). The overall annual improvement rate from 2014 to 2025 was 1.9% (95% CI: 1.0–2.8), suggesting insufficient progress to achieve 80% target by 2030.

### Affordability of essential medicines

#### Overall affordability

**Fig. 5:**
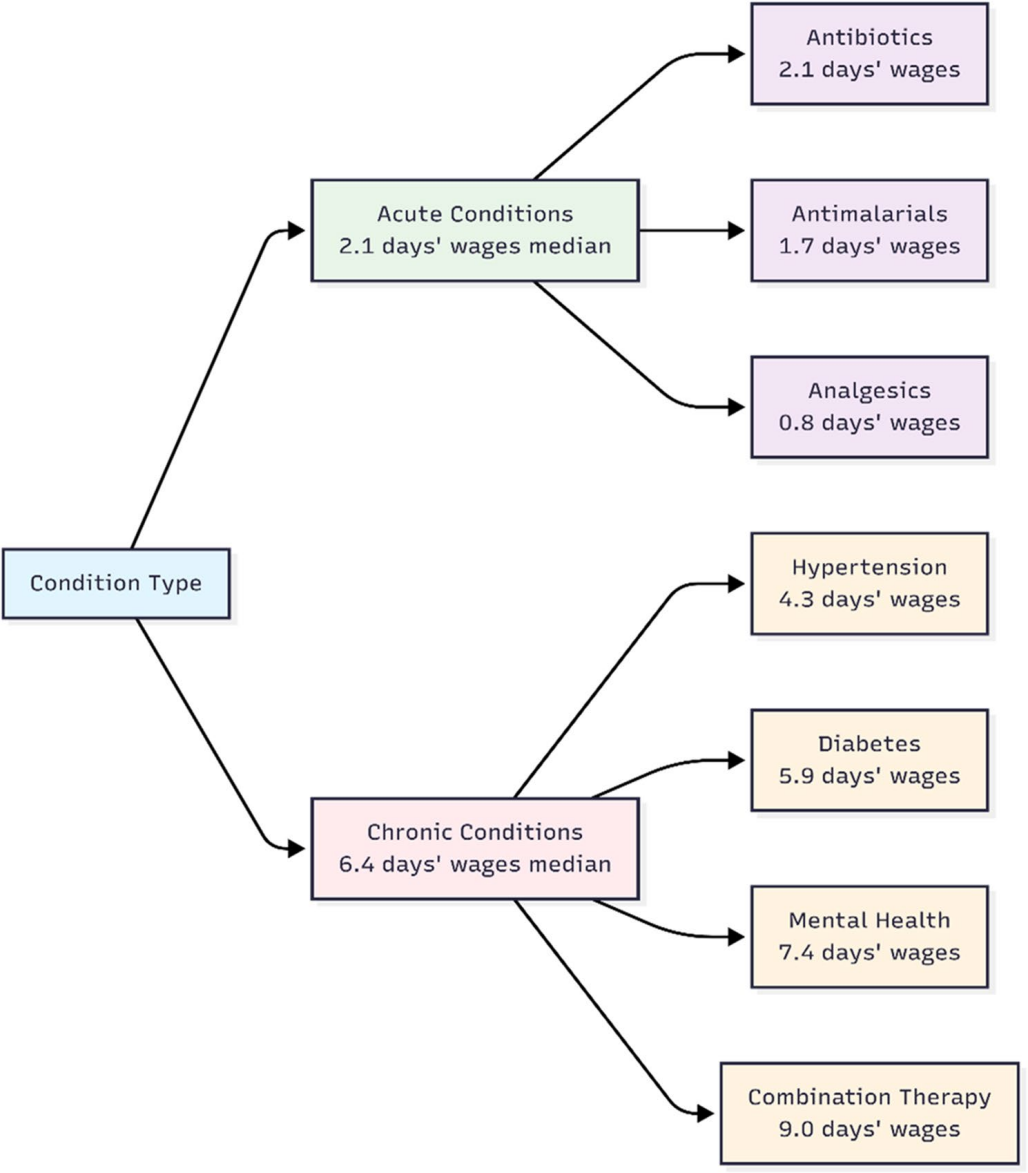
Affordability burden by condition type. Flow diagram showing days’ wages required for acute versus chronic conditions across different therapeutic categories

Treatments for acute conditions required a median of 2.1 days’ wages (IQR: 1.5–3.2) in private sectors, while chronic disease treatments required 6.4 days’ wages (IQR: 4.3–9.1). In public sectors, costs were substantially lower but still exceeded WHO affordability thresholds (> 1 day’s wage) for chronic conditions (Table 4; Fig. 5).

**Table 4:** Affordability analysis by condition type*

Condition Type	Treatment Example	Public Sector (Days’ Wages)	Private Sector (Days’ Wages)	Private: Public Ratio
Acute	Antibiotics (Amoxicillin)	0.4 (0.2–0.7)	2.1 (1.5–3.2)	5.3:1
	Antimalarials (ACT)	0.3 (0.1–0.6)	1.7 (1.1–2.6)	5.7:1
	Analgesics (Paracetamol)	0.1 (0.05–0.3)	0.8 (0.5–1.4)	8.0:1
Chronic	Hypertension (Amlodipine)	1.3 (0.8–2.0)	4.3 (2.9–6.2)	3.3:1
	Diabetes (Metformin)	1.9 (1.3–2.8)	5.9 (4.0–8.3)	3.1:1
	Mental Health (Amitriptyline)	2.5 (1.7–3.6)	7.4 (5.0–10.3)	3.0:1
	Combination Therapy	3.9 (2.6–5.5)	9.0 (6.2–12.5)	2.3:1

•Table 4 Affordability analysis by condition type

•Days' wages required for different treatments in public and private sectors, with private:public cost ratios

*Values are median (IQR). *ACT* Artemisinin-based Combination Therapy

#### Catastrophic health expenditure

Pooled analysis from 33 studies (*n* = 94,217 households) showed 24.1% (95% CI: 20.3–27.9%) of households experienced catastrophic health expenditure (defined as > 10% of household consumption/income) from medicine purchases. Prevalence was higher among chronic disease households (39.8%, 95% CI: 34.1–45.5%) and the poorest wealth quintile (46.3%, 95% CI: 40.7–51.9%).

### Geographical and socioeconomic disparities

Substantial disparities were documented across multiple dimensions (Table 5). The poorest wealth quintile faced 3.3 times higher affordability burden compared to the richest quintile, with availability rates of 35.1% versus 60.2% respectively. Rural areas demonstrated 21.8% points lower availability than urban areas (37.4% vs. 59.2%), and catastrophic health expenditure was nearly twice as prevalent in rural compared to urban households (32.8% vs. 18.9%).

**Table 5:** Geographical and socioeconomic disparities*

Dimension	Availability (%)	Affordability Burden Ratio	Catastrophic Expenditure (%)
Wealth Quintile			
Poorest	35.1	3.3:1	46.3
Richest	60.2	1.0:1	12.5
Residence			
Urban	59.2	1.9:1	18.9
Rural	37.4	4.4:1	32.8
Region			
Central Africa	33.1	5.2:1	42.5
Southern Africa	56.5	2.3:1	19.8

•Table 5 Geographical and socioeconomic disparities

•Comprehensive analysis of availability, affordability burden ratios, and catastrophic expenditure across wealth quintiles, urban-rural divides, and African regions*

All comparisons statistically significant at *p*<0.001. Affordability burden ratio compares poorest to richest quintile; urban to rural; highest burden region to lowest burden region*

### Intervention effectiveness

Analysis of 20 intervention studies revealed varying effectiveness across approaches (Table 6). Supply chain interventions demonstrated the strongest improvements in availability, particularly supply chain digitization (31.7% improvement) and pooled procurement (22.4% improvement). Financing interventions showed the greatest impact on affordability, with social health insurance achieving 32.6% improvement and targeted subsidies 28.4% improvement.

**Table 6:** Intervention effectiveness analysis*

Intervention Category	Specific Intervention	Availability Improvement % (95% CI)	Affordability Improvement % (95% CI)	Evidence Strength
Supply Chain	Pooled Procurement	22.4 (18.3–26.5)	15.8 (12.1–19.5)	Strong
	Supply Chain Digitization	31.7 (26.9–36.5)	18.2 (14.3–22.1)	Moderate
	Integrated Supply Chains	25.7 (21.2–30.2)	12.4 (9.1–15.7)	Moderate
Financing	Social Health Insurance	18.9 (15.1–22.7)	32.6 (28.4–36.8)	Strong
	Targeted Subsidies	15.3 (11.8–18.8)	28.4 (24.3–32.5)	Moderate
	Tax Exemptions	12.7 (9.5–15.9)	21.8 (18.1–25.5)	Moderate
Regulatory	Local Production	16.9 (13.2–20.6)	18.3 (14.7–21.9)	Weak
	Quality Assurance	17.2 (13.6–20.8)	14.6 (11.2–18.0)	Weak
Human Resources	Training Programs	12.3 (9.1–15.5)	8.7 (6.1–11.3)	Weak

•Table 6 Intervention effectiveness analysis

•Summary of evidence for different intervention categories showing percentage improvements in availability and affordability with evidence strength ratings

Evidence Strength ratings: Strong = consistent findings from ≥3 RCTs/multiple robust observational studies; Moderate = consistent findings from observational studies; Weak = limited or inconsistent evidence*

### Sensitivity analysis and publication bias

Sensitivity analysis excluding seven high-risk bias studies yielded similar pooled availability estimates (public: 49.3%, 95% CI: 43.4–55.2; private: 71.2%, 95% CI: 64.8–77.6). Funnel plots for availability meta-analyses showed slight asymmetry, suggesting possible publication bias for smaller studies with extreme effects, but Egger’s test was non-significant (*p* = 0.08).

## Discussion

### Principal findings

This systematic review and meta-analysis demonstrates that essential medicines in African LMICs remain critically unavailable in public sectors and often unaffordable in private sectors. The public sector availability of 48.1% represents a substantial 31.9% point gap from WHO targets, indicating systemic failures in medicine supply systems. Affordability challenges are particularly severe for chronic conditions, requiring over six days’ wages for treatment courses.

### Comparison with existing literature

Our findings align with but extend previous reviews documenting medicine access challenges [13–16]. The therapeutic inequity between communicable (59.1%) and non-communicable (37.4%) disease medicines has widened by approximately 13% points since 2015, reflecting the rapid epidemiological transition without corresponding health system adaptation [8]. The minimal annual improvement rate of 1.9% in public sector availability suggests current approaches are insufficient to achieve medicine-related UHC targets by 2030.

### Interpretation of intervention effectiveness

The intervention analysis provides actionable evidence for policymakers. Supply chain digitization showed the strongest availability improvements (31.7%), likely through enhanced visibility, reduced stock outs, and improved forecasting. Social health insurance demonstrated the greatest affordability impact (32.6% improvement), highlighting the importance of financial risk protection. The weaker evidence for regulatory and human resource interventions suggests these require more robust implementation or combined approaches.

### Policy implications

Four priority policy actions emerge:

6. Supply chain transformation through digital solutions, regional pooled procurement, and strategic local production.
7. Financing reforms including mandatory health insurance expansion, targeted subsidies for vulnerable populations, and increased domestic resource allocation.
8. Regulatory harmonization through the African Medicines Agency to streamline registration, improve quality oversight, and reduce costs.
9. Equity-focused approaches addressing geographical, therapeutic, and socioeconomic disparities through targeted investments and monitoring.

### Strengths and limitations

Strengths include comprehensive searches across six databases (updated to December 2025), rigorous methodology following PRISMA guidelines, independent duplicate review despite single authorship (achieved through staggered screening and independent reviewer consultation), broad geographical coverage (34 countries), and contemporary timeframe including COVID-19 impacts and recovery. The use of random-effects meta-analysis appropriately accounts for heterogeneity.

Limitations include: high statistical heterogeneity (I² >80%) reflecting methodological and contextual diversity; language restrictions to English and French publications; limited data from conflict-affected regions; predominance of cross-sectional studies limiting causal inference; and varying definitions of catastrophic expenditure across studies. The single authorship, while methodologically rigorous through independent verification processes, may still raise concerns about potential bias.

### Future research directions

Future studies should: employ longitudinal designs to assess trends and intervention impacts; include cost-effectiveness analyses of different access strategies; focus on underrepresented regions (Central Africa, conflict zones); examine implementation factors for successful interventions; and develop context-specific equity metrics.

## Conclusion

Essential medicines in African LMICs remain inadequately available and often unaffordable, with significant disparities across regions, therapeutic categories, and socioeconomic groups. The COVID-19 pandemic exposed and exacerbated pre-existing vulnerabilities in medicine supply systems, with only partial recovery by 2025. Achieving meaningful progress requires transformative system-wide reforms addressing supply chain resilience, sustainable financing mechanisms, regulatory harmonization through the African Medicines Agency, and explicit equity focus. As African countries pursue Universal Health Coverage targets, ensuring reliable access to essential medicines must remain a central priority for health system development and a fundamental commitment to health equity.

## Data Availability

All data generated or analysed during this study are included in this published article and its supplementary information files. The datasets used are available from the corresponding author on reasonable request.

## Abbreviations

ACT: Artemisinin-based Combination Therapy
ARVs: Antiretrovirals
CI: Confidence Interval
HAI: Health Action International
IQR: Interquartile Range
LMICs: Low- and Middle-Income Countries
NCDs: Non-Communicable Diseases
PRISMA: Preferred Reporting Items for Systematic Reviews and Meta-Analyses
RCT: Randomized Controlled Trial
SDGs: Sustainable Development Goals
UHC: Universal Health Coverage
WHO: World Health Organization
